# Methods to predict osteonecrosis of femoral head after femoral neck fracture: a systematic review of the literature

**DOI:** 10.1186/s13018-023-03858-7

**Published:** 2023-05-22

**Authors:** Yi Hu, Qin Yang, Jun Zhang, Yu Peng, Qingqing Guang, Kaihu Li

**Affiliations:** 1 Department of Orthopaedics, The First People’s Hospital of Changde City, Changde, China; 2 Department of Hematology, The Third Xiangya Hospital of Central South University, Changsha, China; 3 Department of Orthopaedics, The Second Xiangya Hospital of Central South University, Changsha, China

**Keywords:** Femoral neck fracture, Osteonecrosis of femoral head, Prediction method, Perfusion, Blood supply

## Abstract

**Background:**

Femoral neck fracture (FNF) is a very common traumatic disorder and a major cause of blood supply disruption to the femoral head, which may lead to a severe long-term complication, osteonecrosis of femoral head (ONFH). Early prediction and evaluation of ONFH after FNF could facilitate early treatment and may prevent or reverse the development of ONFH. In this review paper, we will review all the prediction methods reported in the previous literature.

**Methods:**

Studies on the prediction of ONFH after FNF were included in PubMed and MEDLINE databases with articles published before October 2022. Further screening criteria were conducted according to the Preferred Reporting Items for Systematic Reviews and Meta-Analyses. This study highlights all the advantages and disadvantages of the prediction methods.

**Results:**

There were a total of 36 studies included, involving 11 methods to predict ONFH after FNF. Among radiographic imaging, superselective angiography could directly visualize the blood supply of the femoral head, but it is an invasive examination. As noninvasive detection methods, dynamic enhanced magnetic resonance imaging (MRI) and SPECT/CT are easy to operate, have a high sensitivity, and increase specificity. Though still at the early stage of development in clinical studies, micro-CT is a method of highly accurate quantification that can visualize femoral head intraosseous arteries. The prediction model relates to artificial intelligence and is easy to operate, but there is no consensus on the risk factors of ONFH. For the intraoperative methods, most of them are single studies and lack clinical evidence.

**Conclusion:**

After reviewing all the prediction methods, we recommend using dynamic enhanced MRI or single photon emission computed tomography/computed tomography in combination with the intraoperative observation of bleeding from the holes of proximal cannulated screws to predict ONFH after FNF. Moreover, micro-CT is a promising imaging technique in clinical practice.

## Introduction

Femoral neck fracture (FNF) accounts for 53% of all proximal femoral fractures, either causing hip joint dysfunction in young patients or being life-threatening in the elderly [1]. With the increase in traffic accidents and aging, the incidence rate of FNF continues to rise [2]. Globally, hip fractures in people over 50 have doubled in recent years [2, 3]. Fractures of the FNF are unique and have a high possibility of nonunion and osteonecrosis of the femoral head (ONFH), due to the fragile blood supply to the femoral head.

The main source of blood supply to the femoral head comes from the femoral artery and obturator artery. Among them, the most important arterial branch is the medial femoral circumflex artery (MFCA), which originates from either the deep femoral artery (64.6%) or the femoral artery (32.2%) [4]. In 2016, Dewar et al. [5] found that MFCA provided 82% blood supply to the femur head and 67% to the femoral neck. MFCA is extracapsular and becomes the superior, inferior, and posterior retinacular vessels after penetrating the hip joint capsule. Furthermore, the superior retinacular artery at the cervicocephalic junction branches into the superior metaphyseal artery and lateral epiphyseal artery and the latter feeds 70–80% of the femoral head [6, 7]. The lateral epiphyseal artery is eventually anastomosed to the ligament teres artery, a branch from the obturator artery to form an anastomosis system, which is considered to be indispensable to the revascularization of the femoral head after neck fracture [8, 9].

Incidence of ONFH after FNF ranges from 7 to 80% [10–14], with an average rate of about 25% [15–17]. FNF is an important cause of ONFH [18]. ONFH is an avascular bone necrosis disease caused by abnormal blood supply to the femoral head, leading to the ischemic necrosis of bone cells and bone marrow components. Owing to the progressive destruction of bone structure and fracture of the subchondral bone, ONFH eventually would develop into femoral head collapse and secondary hip osteoarthritis, causing severe and recurrent hip pain and loss of joint function [19]. The late-stage ONFH results in serious economic and physical burdens to patients [20].

If ONFH is detected at an early stage before the collapse of the femoral head occurs, hip-preserving therapeutics, which include conservative treatments and hip-preserving operations, could be performed to delay or prevent the occurrence of late-stage ONFH [21]. Biophysical stimulation is the main conservative method, including pulsed electromagnetic field [22] and extracorporeal shock wave therapy [23]. Surgical hip-preserving treatments incorporate osteotomies [24], core decompression [25, 26], and non-vascularized [27] or vascularized bone grafting [28]. Most conservative approaches demonstrate limited effects, while surgical approaches are effective to relieve symptoms and could delay or even prevent the progression of ONFH [29, 30]. Therefore, early prediction and evaluation of ONFH after FNF could make early hip-preserving surgery possible, so they are important ways to reduce the incidence of ONFH with great clinical significance. Many scholars have studied this problem and developed many methods, such as dynamic enhanced magnetic resonance imaging, superselective angiography, Doppler-laser hemodynamic measurement, intraosseous oxygen pressure measurement, and bone scintigraphy. Ehlinger et al. [31] summarized the latest methods in 2011, but in the past decade, numerous new prediction methods have emerged, such as bone SPECT/CT (single photon emission computed tomography/computer tomography), predictive models, and micro-computed tomography scanning, intraosseous artery 3D reconstruction, and quantification. In this review paper, we will systematically update and discuss the methods of predicting ONFH after FNF.

## Materials and methods

### Literature search

We performed a systematic review of the literature on the prediction of ONFH after FNF. PubMed and MEDLINE databases were retrieved using three different search terms: ((femoral neck fracture) AND (blood supply) OR (perfusion) OR (circulation)), (femoral neck fracture) AND (predict), and (femoral neck fracture) and (osteonecrosis of femoral head) OR (necrosis). Similar articles listed below each study and its related citations were explored for additional eligible studies to ensure no studies were missed. The search process was conducted from October 20 to 30, 2022. Records published in English before October 2022 were screened by two independent reviewers (YH, KL) based on the title, abstract, and then, full text to select relevant studies. If there was disagreement between the two reviewers, other co-authors would be consulted.

### Assessment of study quality

All the included studies were evaluated with the modified Critical Appraisal Skills Programme (CASP) by two reviewers (YH, KL). Each study was scored with 12 questions, for which the score was 1 for “Yes” and 0 for “No” or “Can’t tell”. If there was disagreement between the two reviewers, other co-authors would be consulted.

## Results

### Study identification

**Fig. 1:**
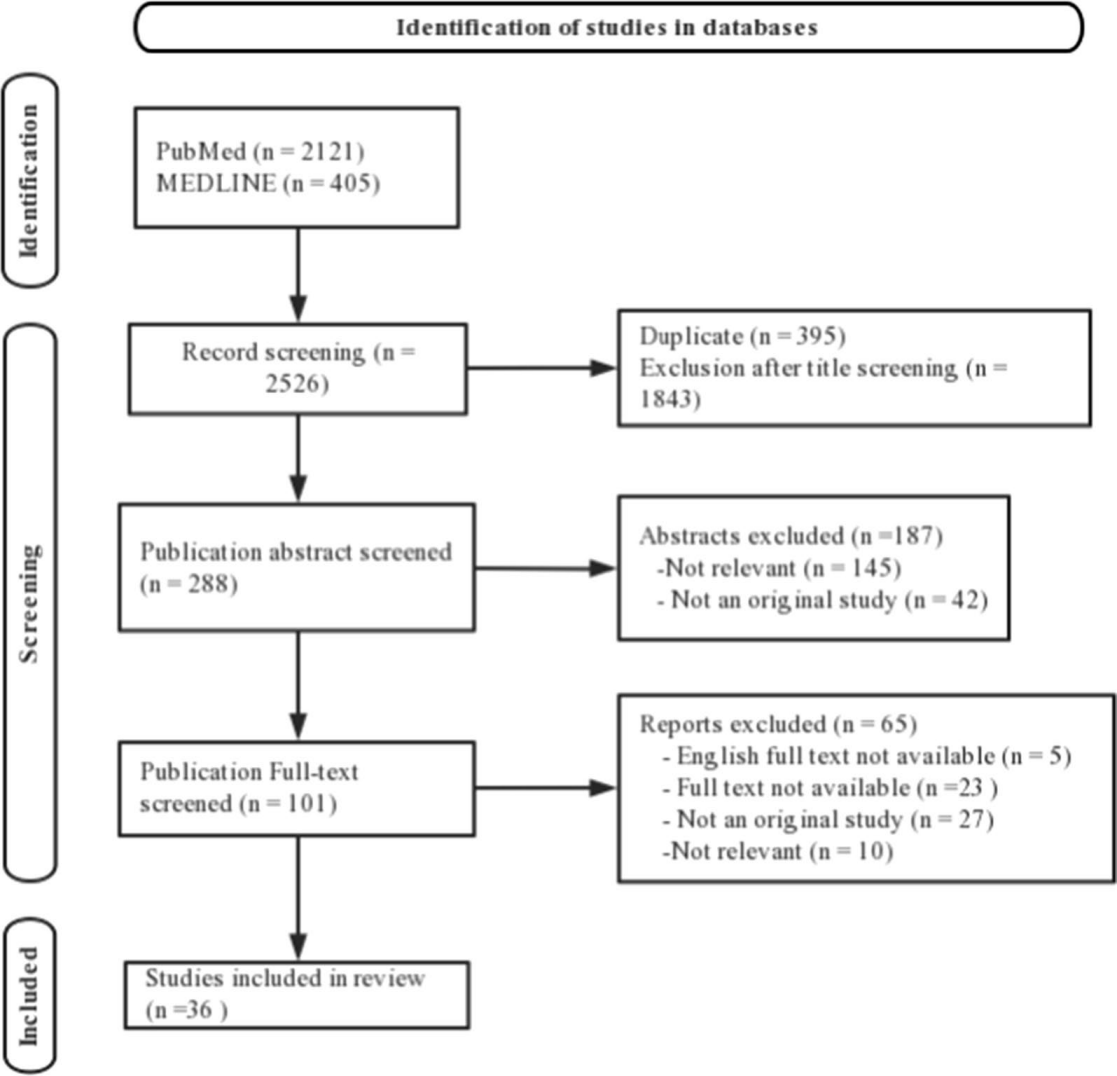
Preferred Reporting Items for Systematic Reviews and Meta-Analyses (PRISMA) flow diagram to screen studies on the prediction methods of osteonecrosis of femoral head (ONFH) after femoral neck fracture (FNF)

A total of 2526 articles were identified in the databases (Fig. 1). After 395 duplicates were removed, 1843 articles were evaluated by title. A total of 1555 articles were excluded because they did not mention femoral neck fracture or femoral head perfusion or prediction. Then, 288 articles were screened by abstract, 187 articles were excluded because 145 articles were not related to the prediction of femoral head perfusion and 42 articles were not original studies. A total of 101 full-text articles were further assessed for eligibility. After a full-text review, 36 articles were included. Sixty-five articles were excluded because 5 articles’ English full text was not available, 23 articles could not get the full text, 27 articles were not an original study, and 10 articles were not related to the prediction of femoral head perfusion.

### Characteristics of the studies

Characteristics of all included studies are shown in Table 1. In total, 2432 cases were included. Ten papers did not indicate the mean age, and 12 papers did not indicate the gender distribution of the cases. The vast majority of patients were female (female/male = 1092/811). For study design, there were 20 prospective studies, 13 retrospective studies, 1 ambispective cohort study, 1 meta-analysis, and 1 paper that did not indicate its study type.

**Table 1:** The detailed parameters of the included studies

Study No	Author	Year	cases	Mean age (years)	Female/Male	Follow-up (months)	Study design	Level	Method to predict ONFH after FNF
*Radiographic imaging*									
1	Jian Xiao et al.	2012	9	44.6	6/3	NR	Prospective	IV	Superselective angiography
2	Yang Liu et al.	2013	45	45.4	19/26	6–60	Prospective	IV	Superselective angiography
3	J. W. Kim et al.	2007	83	67.0	62/21	NR	Retrospective	IV	Bone scintigraphy
4	Ashishkumar K et al.	2018	22	11.2	7/13	NR	Retrospective	IV	Bone scintigraphy
5	Jan Juréus et al.	2016	8	11.5	4/4	> 12	Retrospective	IV	Bone scintigraphy
6	Ji Wan Kim et al.	2017	44	66.9	33/11	24–73	Retrospective cohort	IV	Bone SPECT
7	B. Iqbal et al.	2022	279	NR	NR	NR	Meta-analysis	I	Bone SPECT/CT
8	Sung Jun Park et al.	2019	97	75.4	NR	12–56	Retrospective	IV	Bone SPECT/CT
9	Jae Youn Yoon et al.	2020	30	64.3	19/11	24–106.8	Prospective cohort	III	Bone SPECT/CT
10	Sangwon Han et al.	2017	53	59.1	39/14	12	Retrospective	IV	Bone SPECT/CT
11	Yeon-Hee Han et al.	2019	131	78.0	82/49	NR	Retrospective	IV	Bone SPECT/CT
12	Yoo Sung Song et al.	2022	21	11.0	10/11	> 12	Retrospective	IV	Bone SPECT/CT
13	Heng-feng Yuan et al.	2015	114	58.6	61/53	24	Prospective	III	Bone SPECT/CT
14	Jong Ho Noh et al.	2020	72	54.01	50/22	6–187	Retrospective	IV	Bone SPECT/CT
15	Abhishek Kaushik et al.	2010	31	47	15/15	NR	Prospective	III	Dynamic enhanced MRI
16	Masatoshi Morimoto et al.	2017	64	78	54/14	> 24	Prospective	III	Dynamic enhanced MRI
17	Philipp Lang et al.	1993	20	NR	NR	> 12	Prospective	IV	Dynamic enhanced MRI
18	T. Schneider et al.	2003	30	NR	NR	NR	Prospective	IV	Dynamic enhanced MRI
19	Abhishek Kaushik et al.	2009	31	48	NR	> 6	Prospective	III	Dynamic enhanced MRI
20	Scott N. Nadel et al.	1992	5	NR	NR	NR	Prospective	IV	Dynamic enhanced MRI
21	Dewei Zhao et al.	2017	27	NR	NR	NR	Prospective	IV	Micro-CT
22	Xing Qiu et al.	2016	14	37.2	1/13	NR	NR	IV	Micro-CT
23	Xing Qiu et al.	2018	12	69.5	10/2	NR	Prospective	IV	Micro-CT
*Artificial intelligence*									
24	Huan Wang et al.	2021	259	57	135/124	30	Retrospective	IV	Prediction model
25	Wanbo Zhu et al.	2020	238	> 46.4	106/132	> 60	Retrospective	IV	Prediction model
26	Shuangshuang Cui et al.	2018	120	55	71/49	16–83	Ambispective cohort	III	Prediction model
27	Jiaqi Zheng et al.	2021	378	56	219/159	> 36	Retrospective	IV	Prediction model
*Intraoperative methods*									
28	Gill Thomas J et al.	1998	64	81	34/30	38.4	Prospective	III	Femoral head drilling
29	Jozsef Nyarady et al.	2012	9	NR	NR	29	Prospective	IV	Osteoscopy
30	Yoshinobu Watanabe et al.	2007	18	76.5	13/4	19	Prospective	III	Intramedullary oxygen tension
31	M F Swiontkowski et al.	1987	5	NR	NR	12	Prospective	IV	Doppler-laser flowmetry
32	K Sugamoto et al.	1998	28	NR	NR	NR	Prospective	IV	Doppler-laser flowmetry
33	H P Nötzli et al.	2002	32	NR	NR	NR	Prospective	IV	Doppler-laser flowmetry
34	Myung-Rae Cho et al.	2007	54	51	31/23	25–57	Retrospective	IV	Bleeding from the holes of proximal cannulated screws
35	Tim Schrader et al.	2016	NR	NR	NR	NR	NR	V	Intraosseous pressure
36	Jeffrey Donahue et al.	2021	20	56	11/8	12	Prospective	II	Intraosseous pressure

*NR* Not record

### Literature quality and the risk of bias

Most studies were scored moderately in the field of methodological quality. High-quality randomized controlled trials (RCTs) were rare. The mean score was 7.1, rendering the outcomes accessible to the risk of bias (Table 2). The bias included:

1. Selection bias: Few high-quality RCTs may be the main reason for selection bias, the other source of bias may be inconsistent with patients’ age, and some papers were reported only for children.
2. Performance bias: The bias may exist with no consideration of other factors that can cause ONFH after FNF, particularly intracapsular pressure.
3. Attrition bias: A small number of studies reported outcomes incompletely and exposed considerable loss to follow-up.
4. Detection bias: The fact that most detection results are judged by the professional, different professionals have different results. This may be the main reason for detection bias.
5. Reporting bias: This is an intrinsic weakness of retrospective studies.

**Table 2:** Methodological quality of the included studies based on the modified CASP

References	A focused issue with a question	Method appropriate	Patients recruited appropriately	Define the intervention	Outcomes accurately measured	Confounding factors identified	Confounding factors considered	Follow-up > 80%	Results all clearly defined	Results realistic?	Applicable to the local population	Results proportional to other evidence	Total
Jian Xiao	1	0	0	1	1	0	0	1	1	1	1	1	8
Yang Liu	1	0	0	1	1	0	0	1	1	1	1	1	8
J. W. Kim	1	0	0	0	1	0	0	1	1	1	1	1	7
Ashishkumar K	1	0	0	0	1	0	0	1	1	1	1	1	7
Jan Juréus	1	0	0	0	1	0	0	1	1	1	1	1	7
Ji Wan Kim	1	0	0	0	1	0	0	1	1	1	1	1	7
B. Iqbal	1	1	1	1	0	1	1	C.T	1	1	1	1	10
Sung Jun Park	1	0	0	0	0	0	0	1	1	1	1	1	6
Jae Youn	1	0	0	1	1	0	1	1	1	1	1	1	9
Sangwon	1	0	0	0	0	0	0	1	1	1	1	1	6
Yeon-Hee	1	0	0	0	1	0	0	1	0	1	1	1	6
Yoo Sung	1	0	0	0	0	0	0	1	0	1	1	1	5
Heng-feng	1	0	0	0	1	0	1	1	1	1	1	1	8
Jong Ho	1	0	0	0	1	0	0	1	1	1	1	1	7
Abhishek	1	0	1	0	1	0	0	1	1	1	1	1	8
Masatoshi	1	0	0	0	1	0	0	0	1	1	1	1	6
Philipp	1	0	0	0	1	0	0	1	1	1	1	1	7
T. Schneider	1	0	0	1	1	0	0	0	1	1	0	0	5
Abhishek	1	0	0	0	1	0	0	1	0	1	1	1	6
Scott N	1	0	0	1	1	0	0	0	1	1	0	0	5
Dewei	1	0	0	0	1	0	0	0	1	1	1	1	6
Xing Qiu	1	0	0	1	1	0	0	0	1	1	1	1	7
Xing Qiu	1	0	0	1	1	0	0	0	1	1	1	1	7
Huan Wang	1	0	1	0	1	0	1	1	1	1	1	1	9
Wanbo Zhu	1	0	1	0	1	0	1	1	1	1	1	1	9
Shuangshuang	1	0	1	0	1	0	1	1	1	1	1	1	9
Jiaqi Zheng	1	0	1	0	1	0	1	1	1	1	1	1	9
Gill	1	0	0	1	1	0	0	1	1	1	1	1	8
Jozsef	1	0	0	0	1	0	0	1	1	1	1	1	7
Yoshinobu	1	1	0	1	1	0	0	1	1	1	1	1	9
M F	1	0	0	0	1	0	0	1	0	0	1	1	5
Sugamoto	1	0	0	0	1	0	0	1	0	0	1	1	5
H P	1	0	0	0	1	0	0	1	1	1	1	1	7
Myung	1	0	0	1	1	0	0	1	1	1	1	1	8
Tim	1	0	0	0	0	0	0	0	1	1	1	1	5
Jeffrey	1	1	0	1	0	0		1	1	1	1	1	8

*C.T* cannot tell

### Superselective angiography

Superselective angiography is a kind of digital subtraction arteriography (DSA) first proposed by Théron [32] in 1977 to assess femoral head vascularity. It can dynamically observe the femoral head vascularity by injecting a superselective contrast agent into the femoral artery. We could directly see the blood supply to the femoral head and indirectly predict the risk of ONFH after FNF by angiography imaging results. Langer et al. [33] conducted research on femoral head vascularity by applying superselective angiography in 35 normal subjects and nine patients with FNFs and found that MCFA was interrupted or rarely existed in 93% of cases of ONFH. He concluded that superselective angiography was a very good method to predict ONFH. Liu et al. [34] sorted patients with FNF by preoperative superselective angiography as follows: Type I: three-six supporting vessels through fracture line; Type II: one-two supporting vessels through fracture line; Type III: no supporting vessels through fracture line. The higher classification was found to have a higher incidence of femoral head necrosis at the follow-up of 6–60 months. Patients with Type I and Type II were advised to take early reduction and internal fixation operations. And patients with Type III were advised to opt for a hip replacement or other early intervention to increase blood supply to the femoral head to avoid necrosis afterward. Superselective angiography is a direct method to observe the blood distribution in the femoral head without severe pain during the examination. Orthopaedic surgeons can intervene timely and provide personalized surgery for patients with FNF according to the results of the angiography examination. Since angiography is invasive, there are some disadvantages, such as risks of arterial dissection, thrombosis, and hematoma formation.

### Bone scan imaging

Bone scan imaging is a diagnostic technique determined by the radioactive tracer uptake at the lesion site after intravenous injection of radioactive tracer, including bone scintigraphy, bone SPECT (single photon emission computed tomography), and bone SPECT/CT. The absorption of radioactive tracer is mainly affected by the local blood supply and mineral metabolism [7]. The FNF is prone to damage the main femoral head vessels, resulting in low perfusion and low metabolic activity, and ultimately ONFH. In bone scan imaging, Images with reduced radioactive tracer uptake in the femoral head could be used to predict ONFH.

The application of bone scan imaging in the prediction of ONFH has undergone a long process. In 1950, Tucker [35] began using a radioactive tracer to diagnose ONFH, and then, Riggins [36] first reported using bone scintigraphy in 1974. Bone scintigraphy imaging is a single-plane imaging, showing only the anterior and posterior positions of the pelvis, which was under suspicion to predict ONFH after FNF. Kim et al. [37] conducted a retrospective analysis of preoperative bone scintigraphy imaging of 83 patients with FNFs and their Garden classification, finding that radioactive tracer uptake in the femoral head after FNF usually corresponded to the degree of fracture displacement. Whereas, preoperative bone scintigraphy seemed to be redundant in the choice of surgical treatment for patients with FNFs. Later, bone SPECT and bone SPECT/CT appeared, respectively. Though bone SPECT imaging is multi-plane, it could not provide accurate anatomical information. Bone SPECT/CT is widely used because of its multi-planar and precise location characteristics nowadays, which is performed by using a hybrid dual-head gamma camera in combination with CT multi-plane 3D (three-dimensional) imaging. It has the advantages of being noninvasive, unaffected by metal implants, and highly sensitive, whereas its disadvantages are poor specificity, and long examination time that some patients cannot tolerate. Another important disadvantage, which reduces the accuracy of its detection, is that it is difficult to quantify and relies on a subjective judgment from experts.

In the past decades, studies have developed many different methods to avoid the shortcoming of non-quantification in SPECT/CT [38–40]. Preoperative quantitative indicators included a percentage of photon defect [41], head-to-head uptake ratio or head-to-acetabulum uptake ratio [42], and the ratio of radionuclide uptake of the affected fractured femoral head to the healthy contralateral femoral head (F/N) [43, 44]. Postoperative quantitative indicators include the mean standardized uptake value of the femoral head (SUV) [45, 46]. Park et al. [41] concluded that the percentage of photon defect in the femoral head was a useful index to determine the surgical methods for FNFs, considering hip replacement when the photon defect was greater than 15%, and internal fixation if the photon defect was less than 15%. Yoon et al. [42] proposed that the critical value of the head-to-head uptake ratio was 0.5 and the critical value of the head-to-acetabulum uptake ratio was 0.3, which could be used as a predictive threshold of ONFH after trauma. Yuan et al. [43] recommended hip replacement when F/N was less than or equal to 0.5 and suggested hollow screw fixation was a more appropriate treatment if F/N ratio is greater than 0.5. Song et al. [45] concluded that patients with a lower mean SUV ratio were highly likely to develop femoral head necrosis in the future.

In recent 5 years, there has been a lot of literature on bone SPECT/CT to predict the prognosis of patients with FNF. Iqbal et al. [47] included seven studies to conduct a meta-analysis on bone SPECT/CT, indicating the prediction of ONFH by bone SPECT/CT is reliable with a 94% sensitivity and a 75% specificity.

### Prediction models of ONFH after FNF

The prediction model, also known as risk score, is a quantitative technique for risk assessment, which has been widely applied in the field of traumatic orthopedics [48]. Prediction models of ONFH after FNF are built through a series of sequential steps consisting of candidate risk factors selection, sample data preprocessing, data balancing, variables selection, parameters modeling and adjusting, and model fitting effect evaluation. The process of prediction models could not be completed without the help of artificial intelligence, which includes multivariable regression and machine learning. And machine learning plays the main role in the prediction model.

In 2018, Cui et al. [49] conducted a cohort study on 120 patients with FNF undergoing closed reduction and cannulated screw internal fixation. In the study, his team established The Naive Bayes Classifier by machine learning with parameters of age, gender, mechanism of injury, side of fractures, preoperative traction, Pauwels angle, and three spatial parameters of displacement, which were measured on preoperative CT scans using three-dimensional software. However, its accuracy (74.4%), sensitivity (74.2%), specificity (75%), positive predictive value (92%), negative predictive value (42.9%), and area under curve (AUC) (0.746) were not very convincing. Later on, a convolutional neural network (CNN) model [50] and a risk score [51] emerged subsequently, which made great progress on the validation set, but without external validation. The CNN model was trained using postoperative pelvic radiographs and the output regressive radiograph variables. The accuracy rose to 0.873, and the AUC reached 0.912 in the prediction of a two-center retrospective study including 238 patients with FNF undergoing closed reduction and cannulated screw fixation surgery[50]. The risk score was constructed by Cox regression analysis in a form of nomogram, which included Garden alignment index, time to surgery, preoperative displace, impaction, and postoperative malposition. In the development and validation cohort, the concordance index was 0.96 and 0.94; and the discrimination slope was 0.51 and 0.47 [51]. Wang et al. [52] proposed a six-variable XGBoost model by comparing different models, established by machine learning algorithms, in predicting ONFH after FNFs treated with internal fixation. They concluded that the six-variable XGBoost model with six predictors, including reduction quality, VAS score, Garden classification, operative time, cause of injury, and fracture location, could better predict the risk of ONFH. Moreover, this model can be generalized to external data, like clinical practice to evaluate the incidence of ONFH after FNF. In the six-variable XGBoost model, the accuracy, sensitivity, and AUC on the validation set were 0.987, 0.929, and 0.992, respectively. On external data, its accuracy, sensitivity, specificity, and AUC were 0.907, 0.807, 0.935, and 0.933, respectively. Meanwhile, the log-loss in the model was only 0.279.

Positively, the prediction model is noninvasive, easy to operate, and better at predicting ONFH after FNF. However, the existing prediction models still have some unsolved shortcomings. There is no consensus on the risk factors of ONFH, and most of them are from retrospective studies. Further research is still needed in the future to improve and optimize these prediction models.

### Magnetic resonance imaging (MRI)

MRI is a noninvasive and effective diagnostic technique for many musculoskeletal disorders. In ONFH, MRI can detect early changes in the femoral head. When the ONFH occurs, the signal of MRI inside the femoral head presents an annular, focal, or diffuse weakened lesion [53]. Though MRI could provide precise information about the morphologic features in the femoral head, it does not show its vascularity and is inadequate to assess bone cell viability in the early post-traumatic phase. Currently, scholars agree that the interval between microstructure changes in the femoral head and positive findings on MRI is long and the specific time is controversial. Asnis et al. [54] studied 20 patients with FNFs undergoing hip replacement and found no significant changes in histology and MRI signaling in isolated femoral heads over a long period. He concluded that MRI could not predict ONFH within 2 weeks following FNF. Kawasaki et al. [55] performed MRI in 31 patients with FNF at 2, 6, and 12 months after surgery and proposed that the most sensitive, specific, and accurate time interval for MRI diagnosis of ONFH was 6 months after surgery. In an animal experiment, Nakamura et al. [56] hypothesized that the minimum interval for MRI to detect ONFH was about 4 weeks after injury.

To show vascularity and find early changes of ONFH, dynamic enhanced MRI has been developed, which may allow detection of blood flow and is useful for noninvasive evaluation of femoral head perfusion after intravenous injection of contrast agent [57, 58]. Cova [59], Nadel [60], and Schneider [61] established animal models to demonstrate that dynamic enhanced MRI can detect femoral head blood flow based on local signal changes. Thereafter, it was gradually applied to patients with FNF and classified according to dynamic curves [62–64] and positive enhancement integral color mapping (PEICM) [65], to guide clinical diagnosis and treatment. Hirata et al. [62] conducted a prospective study on 36 patients with FNF taking dynamic enhanced MRI within 48 h after injury, which was then divided into three groups as follows: Group A, femoral head perfusion was normal; Group B, partial perfusion impairment; Group C, femoral head without perfusion. All patients in Group A and Group B were cured, while ten of 19 patients in Group C showed ONFH eventually. Kaushik et al. [63, 64] found dynamic enhanced MRI can be used to predict the vascular status of the femoral head after FNF because of the reliability of dynamic curve A and B. Morimoto et al. [65] estimated femoral head perfusion before surgery through dynamic enhanced MRI PEICM and divided it into three categories: Type A, the same color as the healthy contralateral side, indicating normal perfusion; Type B, darker than the contralateral side, indicating reduced perfusion; Type C, dark black color, indicating no perfusion at all. According to the classification, patients with a complete lack of femoral head perfusion (Type C) should be treated with hip replacement or hip resuscitation with increased local blood supply, while internal fixation is recommended for patients with normal blood supply or partial perfusion impairment.

The studies above indicate dynamic enhanced MRI is an effective and accurate method to assess the femoral head vascularity after FNF [58, 66]. However, it is relatively expensive and inappropriate to be applied in patients with metal fixators, renal dysfunction, or claustrophobia.

### Micro-computed tomography (Micro-CT)

Micro-CT has emerged as a high-resolution imaging method that can analyze structures with a pixel size on the order of ten μm. The perfusion of the barium sulfate suspension followed by micro-CT scanning can reconstruct and quantify the femoral head intraosseous arteries, clearly demonstrating their anastomoses. With special software programs, we can observe the diameter, length, volume, and density of the blood vessels in the femoral neck and head.

In 2016, micro-CT was used by Qiu et al. [67] to visualize femoral head intraosseous arteries in 14 fresh lower limbs of Chinese cadavers, which can deliver high-resolution 3D digitized data and images of intraosseous arteries by intravenously perfusing the barium sulfate suspension. Later, Zhao et al. [68] applied this method in evaluating the residual blood supply of the femoral head in 27 patients with FNFs before surgery. By digital sub-traction angiography analysis, data indicated that the inferior retinacular arterial system had the highest possibility to be unaffected after FNF with 100% (14 out of 14) in nondisplaced fractures and 60% (six out of ten) in Garden Type III fractures. In the experiment conducted by Qiu et al. [69], 12 femoral head specimens following hip replacement were perfused by micro-CT scanning, and the arterial 3D reconstructions were performed, demonstrating micro-perfusion of the femoral head through the inferior retinacular arteries were feasible. The epiphyseal arterial network and its fine artery branches can be presented in pathologic conditions, which can provide a morphological basis for the study of femoral head diseases.

Micro-CT has been widely used for highly accurate quantification of the tumor's 3D vascular network, coronary arteries, and the entire vasculature of the brain. However, its application in the prediction of ONFH after FNF needs further investigation because only a few clinical studies have been performed.

### Intraoperative methods to predict ONFH after FNF

#### Femoral head drilling during an operation

In 1998, Gill et al. [70] conducted a prospective study of 64 patients with FNF and developed an intraoperative method for predicting ONFH after internal fixation. During the operation, two to four holes were drilled with a 2.0 mm drill at the base of the femoral head fragment with three-four mm space between each hole to observe the bleeding from the femoral head. The conclusion was that its sensitivity and specificity in predicting ONFH after FNF were 100%. There are some unsolved questions. For example, no consensus has been achieved on the threshold of bleeding from the femoral head. Additionally, arterial blood pressure and underlying vascular diseases vary among each patient, so it is hard to standardize the status of intraoperative bleeding in the drilled holes.

#### Osteoscopy

In 2012, NyaradyJ et al. [71] developed a minimally invasive endoscopic system, namely osteoscopy, capable of estimating femoral head circulation. They classified it into four categories according to the intraosseous bleeding and the pressure difference between systolic blood pressure and saline pressure during operation as follows: (1) excellent circulation: when the pressure difference was no more than 30 mmHg at the first sign of diffuse bleeding or the osteoscopy revealed a ‘‘pulsatile’’ bleeding; (2) average circulation: the pressure difference was between 30 and 60 mmHg at the first appearance of diffuse bleeding from the femoral head; (3) minimal circulation: the difference between was more than 60 mmHg; (4) no circulation: no bleeding was detected in the femoral head during osteoscopy. The pressure difference between systolic blood pressure and saline pressure represents femoral head vascular perfusion capacity. The smaller the difference, the stronger the perfusion capacity. Osteoscopy can provide accurate and detailed information about the femoral head circulation during operation, but it could not be sufficiently quantified for routine clinical use. Moreover, pressure detection needs to be refined and its efficacy needs to be confirmed in more patients.

#### Measurement of intramedullary oxygen tension

Watanabe et al. [72] first proposed to predict ONFH by measuring intramedullary oxygen tension in the femoral head and neck during an internal fixation operation in 2007. The subchondral and osteal oxygen tension in the diseased hip was significantly lower than in the normal hip [73]. The difference in intramedullary oxygen tension between the central and peripheral regions of the femoral head is predictive of the occurrence of ONFH after FNF. By using polarographic oxygen electrodes and an oxygen monitor, the intramedullary oxygen tension was measured at four points: (A) one cm distal from the joint surface; (B) one cm proximal from the fracture site; (C) one cm distal from the fracture site; and (D) one cm proximal from the lateral wall. The presence or absence of ONFH was evaluated by MRI at 2, 6, and 12 months after surgery. They found that in patients who developed ONFH, the oxygen tension of point A was lower than point B and the cut-off value was set at 3.1 mmHg. The sensitivity and specificity of this prediction method were 1.0 and 0.82. However, only 17 patients were included in the study.

#### Doppler-laser hemodynamic measurement

Doppler-laser flowmetry was first used by Swiontkowski et al. [74] to evaluate the blood flow in the femoral head. Intraoperatively, a small hole was drilled into the femoral head using a burr, and a probe was inserted to measure intramedullary blood flow. In a study of 32 patients undergoing hip joint surgery, Notzli et al. [75] used Doppler-laser flowmetry to assess the femoral head blood flow, which proved to be useful in clinical observation. Sugamoto et al. [76] applied Doppler-laser in 28 patients with FNFs and found that high flow measurements and sinusoidal waves in shape with the same frequency as the heart rate represented a normal flow distribution in the femoral head, while low flow measurements and non-sinusoidal waves meant ischemia distribution. Doppler-laser hemodynamic measurement is a direct way to evaluate the femoral head blood flow. However, the equipment is costly and the detection is invasive.

#### Bleeding from the holes of proximal cannulated screws

In 2007, Myung-Rae et al. [77] proposed a simple way for intraoperative evaluation of femoral head blood supply by observing the blood drainage from the heads of the cannulated screws used in FNFs fixation for fix minutes. In their study, 44 patients with FNFs were analyzed retrospectively with an average follow-up of 39 months. The patients were classified into two groups: the bleeding group (38 cases with one case of ONFH), and the nonbleeding group (six cases with six cases of ONFH). For the bleeding observation method, the sensitivity, specificity, positive predictive value, and negative predictive value were 86%, 100%, 100%, and 97%, respectively. However, bleeding is a subjective judgment, which cannot change the surgery procedure since the fixation method has been decided preoperatively.

#### Measurement of intraosseous pressure by intracranial pressure monitor

In 2016, Schrader et al. [78] used intracranial pressure (ICP) for the first time to monitor epiphyseal perfusion in patients with slipped capital femoral epiphysis (SCFE). Recently, Donahue et al. [79] applied it to monitor intraoperative femoral head perfusion pressure in 19 adults with FNFs. The ICP monitor probe was inserted into the femoral head to record the intraoperative femoral head perfusion pressure and waveforms. It was believed that waveforms with measurable arterial-like pulsations, synchronous with the heart rate of the patient, represented femoral head perfusion. If the patient with FNF did not have a waveform, surgeries to restore femoral head perfusion would be performed, such as joint capsule decompression and vascular bone flap transplantation. The measurement of intraosseous pressure by ICP monitor can guide the decision-making of surgery. Moreover, it is inexpensive, minimally invasive, and easy to operate. However, the current clinical evidence is insufficient, and further studies are needed to validate its role in a long term.

## Discussion

To summarize, the above methods for predicting ONFH in patients with FNF are effective, but each one has its advantages and disadvantages (Table 3). Superselective angiography is an invasive procedure depending on individual skills and experiences. The doctors inevitably risk exposing to radiation. In the prediction models, the risk factors of ONFH after FNF have not been fully confirmed. Some intraoperative prediction methods require instruments and equipment, such as Doppler-laser hemodynamic measurement, osteoscopy, measurement of intraosseous pressure by LCP, and measurement of intramedullary oxygen tension, which could be used as auxiliary approaches if the devices are accessible during operations. If closed reduction could be achieved in an FNF, then bleeding from the holes of proximal cannulated screws could be applied to predict ONFH. Since no incision is needed, it is convenient to change the surgical procedure according to the prediction results. For FNF patients with open reduction, the femoral head could be exposed and femoral head drilling is preferable. After comprehensive consideration, we recommend selecting dynamic enhanced MRI or SPECT/CT in combination with the intraoperative prediction of bleeding from the holes of proximal cannulated screws to predict ONFH after FNF. Both dynamic enhanced MRI and SPECT/CT can qualitatively and quantitatively evaluate femoral head perfusion in patients with FNF, which have a high sensitivity and specificity [41–45, 62–65]. However, dynamic enhanced MRI and SPECT/CT could not be performed in an emergency case, or on weekends. If we follow the early internal fixation principle for patients with FNF, then it is favorable and valuable to apply the intraoperative approaches, like bleeding from the holes of proximal cannulated screws. Micro-CT seems to be a promising method in the future. Though its clinical application has not been widely carried out, we think it is worth trying in the future.

**Table 3:** Methods to predict osteonecrosis of femoral head (ONFH) after femoral neck fracture (FNF)

Prediction method	Advantages	Disadvantages
*Radiographic imaging*		
Superselective angiography	Direct vascular visualization	Invasive examination, arterial dissection, thrombosis, and hematoma formation
SPECT/CT	Noninvasive, unaffected by metal implants, and high sensitivity	Poor specificity, long time for examination, subjective judgment
Dynamic enhanced MRI	Noninvasive, easy to operate	No consensus on the risk factors of ONFH
Micro-CT	Noninvasive, high specificity and sensitivity	Expensive, inappropriate to be applied in patients with metal fixators, renal dysfunction, or claustrophobia
*Artificial intelligence*		
Prediction model	Femoral head intraosseous arteries visualization, highly accurate quantification	Insufficient clinical evidence
*Intraoperative methods*		
Femoral head drilling	Simple, convenient	Subjective judgment, many confounding factors
Osteoscopy	Direct	Hard to quantify, invasive
Intramedullary oxygen tension	Simple	Insufficient clinical evidence, invasive
Doppler-laser flowmetry	Direct	Costly, insufficient clinical evidence, invasive
Bleeding from the holes of proximal cannulated screws	Simple, convenient	Subjective judgment
Intraosseous pressure	Easy to operate	Insufficient clinical evidence, invasive

**Fig. 2:**
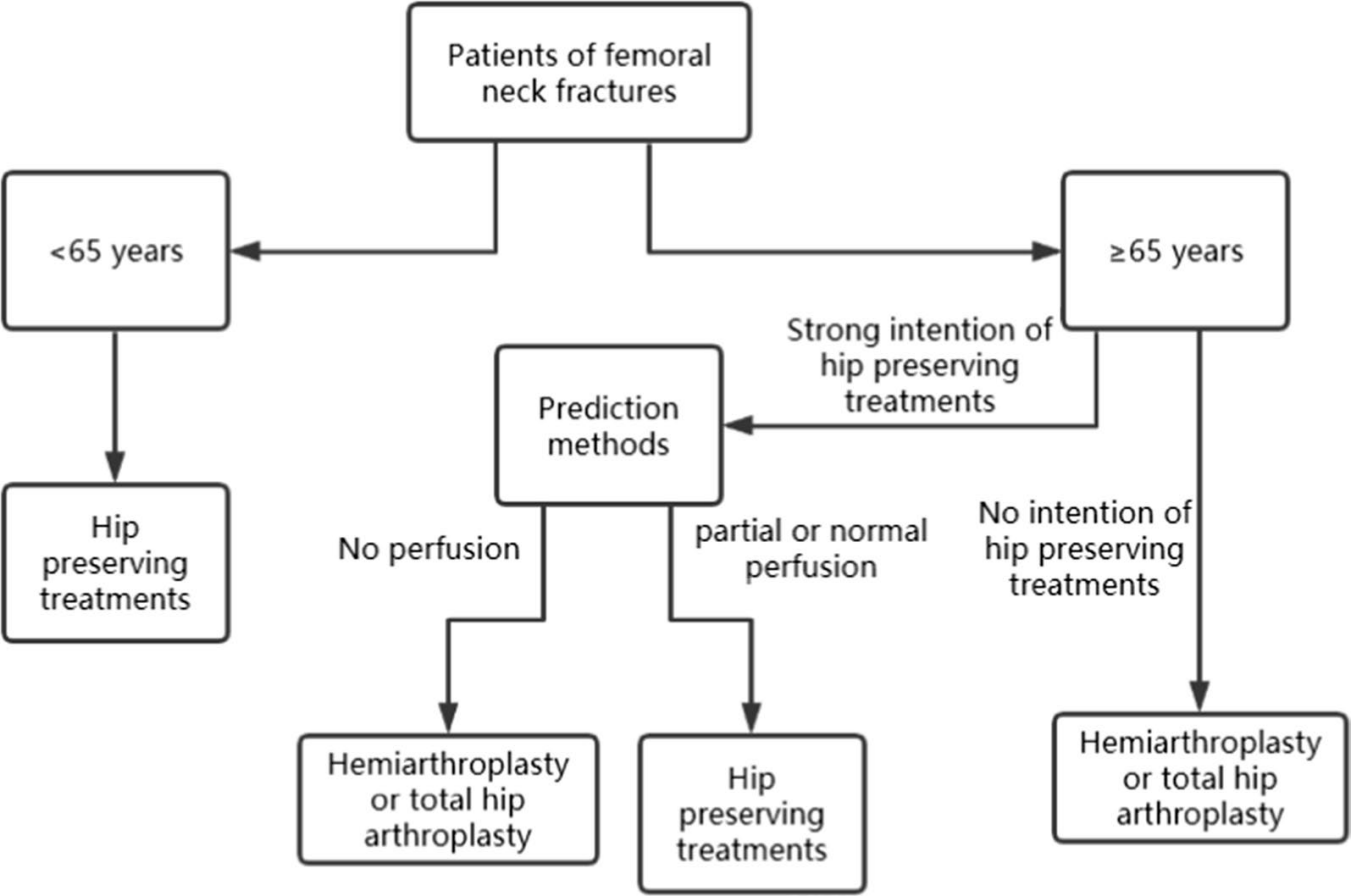
The treatment flowchart of femoral neck fractures (FNFs)

In clinical work, we recommend hip-preserving treatments for patients younger than 65 years old [80]. For patients older than 65 years having a strong intention to preserve their hip joints, the appropriate methods to predict ONFH should be performed quickly. According to results from the prediction methods of ONFH after FNF, we can provide personalized therapeutics for the patients. Hemiarthroplasty or total hip arthroplasty should be performed if no perfusion is detected in the femoral head, and hip-preserving treatments are recommended if the femoral head has partial or normal perfusion [81]. However, we cannot ignore the life-threatening complications in the beds for the elderly after the hip-preserving operation. Therefore, we should make a comprehensive judgment based on the patient’s physical condition. The flowchart of clinical recommendations is shown in Fig. 2.

## Conclusion

After reviewing all the prediction methods, we recommend using dynamic enhanced MRI or SPECT/CT in combination with an intraoperative prediction of bleeding from the holes of proximal cannulated screws to predict ONFH after FNF. In addition, micro-CT is a promising imaging technique in clinical practice. However, the selection of prediction methods for a patient with FNF should be based on his or her situation.

## Data Availability

Not applicable.

## Abbreviations

FNF: Femoral neck fracture
ONFH: Osteonecrosis of femoral head
PRISMA: Preferred Reporting Items for Systematic Reviews and Meta-Analyses
MRI: Magnetic resonance imaging
SPECT/CT: Single photon emission computed tomography/computed tomography
MFCA: Medial femoral circumflex artery
DSA: Digital subtraction arteriography
3D: Three-dimensional
F/N: The ratio of radionuclide uptake of the affected fractured femoral head to the healthy contralateral femoral head
SUV: The mean standardized uptake value of femoral head
AUC: Area under curve
CNN: Convolutional neural network
PEICM: Positive enhancement integral color mapping
Micro-CT: Micro-computed tomography
ICP: Intracranial pressure
SCFE: Slipped capital femoral epiphysis
